# Adult murine cardiomyocytes exhibit regenerative activity with cell cycle reentry through STAT3 in the healing process of myocarditis

**DOI:** 10.1038/s41598-017-01426-8

**Published:** 2017-05-03

**Authors:** Akimitsu Miyawaki, Masanori Obana, Yusuke Mitsuhara, Aya Orimoto, Yusuke Nakayasu, Tomomi Yamashita, So-ichiro Fukada, Makiko Maeda, Hiroyuki Nakayama, Yasushi Fujio

**Affiliations:** 1 Laboratory of Clinical Science and Biomedicine, Graduate School of Pharmaceutical Sciences, Osaka University, 1-6 Yamadaoka, Suita, Osaka 565-0871 Japan; 2 Laboratory of Molecular and Cellular Physiology, Graduate School of Pharmaceutical Sciences, Osaka University, 1-6 Yamadaoka, Suita, Osaka 565-0871 Japan

## Abstract

Mammalian cardiomyocytes substantially lose proliferative capacity immediately after birth, limiting adult heart regeneration after injury. However, clinical myocarditis appears to be self-limiting with tissue-reparative properties. Here, we investigated the molecular mechanisms underlying the recovery from myocarditis with regard to cardiomyocyte proliferation using an experimental autoimmune myocarditis (EAM) model. Three weeks after EAM induction (EAM3w), cardiac tissue displayed infiltration of inflammatory cells with cardiomyocyte apoptosis. However, by EAM5w, the myocardial damage was remarkably attenuated, associated with an increase in cardiomyocytes that were positively stained with cell cycle markers at EAM3w. Cardiomyocyte fate mapping study revealed that the proliferating cardiomyocytes primarily derived from pre-existing cardiomyocytes. Signal transducer and activator of transcription 3 (STAT3) was robustly activated in cardiomyocytes during inflammation, accompanied by induction of interleukin-6 family cytokines. Cardiomyocyte-specific ablation of *STAT3* gene suppressed the frequency of cycling cardiomyocytes in the recovery period without influencing inflammatory status, resulting in impaired tissue repair and cardiac dysfunction. Finally, microarray analysis revealed that the expression of regeneration-related genes, *metallothioneins* and *clusterin*, in cardiomyocytes was decreased by *STAT3* gene deletion. These data show that adult mammalian cardiomyocytes restore regenerative capacity with cell cycle reentry through STAT3 as the heart recovers from myocarditis-induced cardiac damage.

## Introduction

Mammalian cardiomyocytes exit from the cell cycle immediately after birth^1, 2^. Therefore, the proliferative capacity of cardiomyocytes is limited in adult mammals, explaining the etiology of heart failure. For instance, in ischemic insults, dead cardiomyocytes are replaced predominantly with fibrotic tissue, not with proliferating cardiomyocytes, resulting in impaired contractility^3, 4^. Thus, cardiac homeostasis in adult mammals has been believed to be maintained mainly by protection of cardiomyocytes rather than by their proliferation.

In this context, a number of efforts have been made to identify cardioprotective factors to develop novel therapeutic strategies. Accumulating evidence has revealed that signal transducer and activator of transcription 3 (STAT3) is a potent cardioprotective factor^5^. STAT3 is phosphorylated at Y705 by Janus kinase (JAK) upon interleukin-6 (IL-6) family cytokine stimulation^6^, and phosphorylated STAT3 is translocated to the nucleus to activate transcription of anti-oxidant and anti-apoptotic molecules, such as metallothioneins^7–9^ and bcl-xL^10^; however, no evidence that STAT3 functions as a proliferative factor in adult mammalian cardiomyocytes has been proposed due to their low proliferative/regenerative capacity.

In contrast, the involvement of STAT3 in cardiomyocyte proliferation has been addressed exclusively in zebrafish and neonatal mouse hearts^11, 12^, because zebrafish and neonatal mouse cardiomyocytes, unlike adult mammals, possess the proliferative capacity and respond to trauma by reentering the cell cycle^13–17^. Importantly, when cardiac STAT3 is inhibited by its dominant negative form, cardiomyocyte proliferation after ventricular amputation in zebrafish is decreased by ~80%, resulting in insufficient heart regeneration^11^. Recently, it has also been documented that STAT3 is required for regeneration of neonatal mouse hearts by using ventricular amputation model^12^, while ventricular dissection results in cardiac scar formation without repair in adult mouse hearts^15^. It should also be noted that STAT3 is activated in post-infarct myocardium but that STAT3 activation fails to induce cardiomyocyte proliferation at significant frequency in adult mouse hearts^8, 15, 17, 18^, though myocardial activation of STAT3 contributes to cardioprotection^7–9^ and angiogenesis^19, 20^, leading to prevention of adverse cardiac remodeling.

In contradiction to the limited regenerative capacity of adult mammalian hearts, it is well known in clinical settings that most patients with myocarditis, who temporarily exhibit cardiac dysfunction, display spontaneous recovery after acute inflammation is ceased^21, 22^. Therefore, it is conceivable that adult mammalian hearts show healing capability from injury in myocarditis, although the cellular and molecular mechanisms underlying the recovery process are poorly understood.

In the present study, to clarify the endogenous reparative activities observed in myocarditis, we employed experimental autoimmune myocarditis (EAM) as a murine myocarditis model^23, 24^. Similar to human myocarditis, we found that EAM spontaneously relented and that substantial proportion of cardiomyocytes reentered the cell cycle in the process of tissue restoration. Cardiomyocyte fate mapping study revealed that the proliferating cardiomyocytes were derived from pre-existing cardiomyocytes, rather than precursor or stem cell population. STAT3 was robustly activated in the inflamed heart and promoted tissue restoration as a cytoprotective and proliferative factor. This is the first demonstration that activation of STAT3 plays important roles in the myocardial recovery from myocarditis-induced damage in adult mammalian hearts, providing mechanistic insights into the self-limiting nature of myocarditis.

## Results

### Cardiac tissue was restored from inflammation-induced injury through EAM resolution

**Figure 1:**
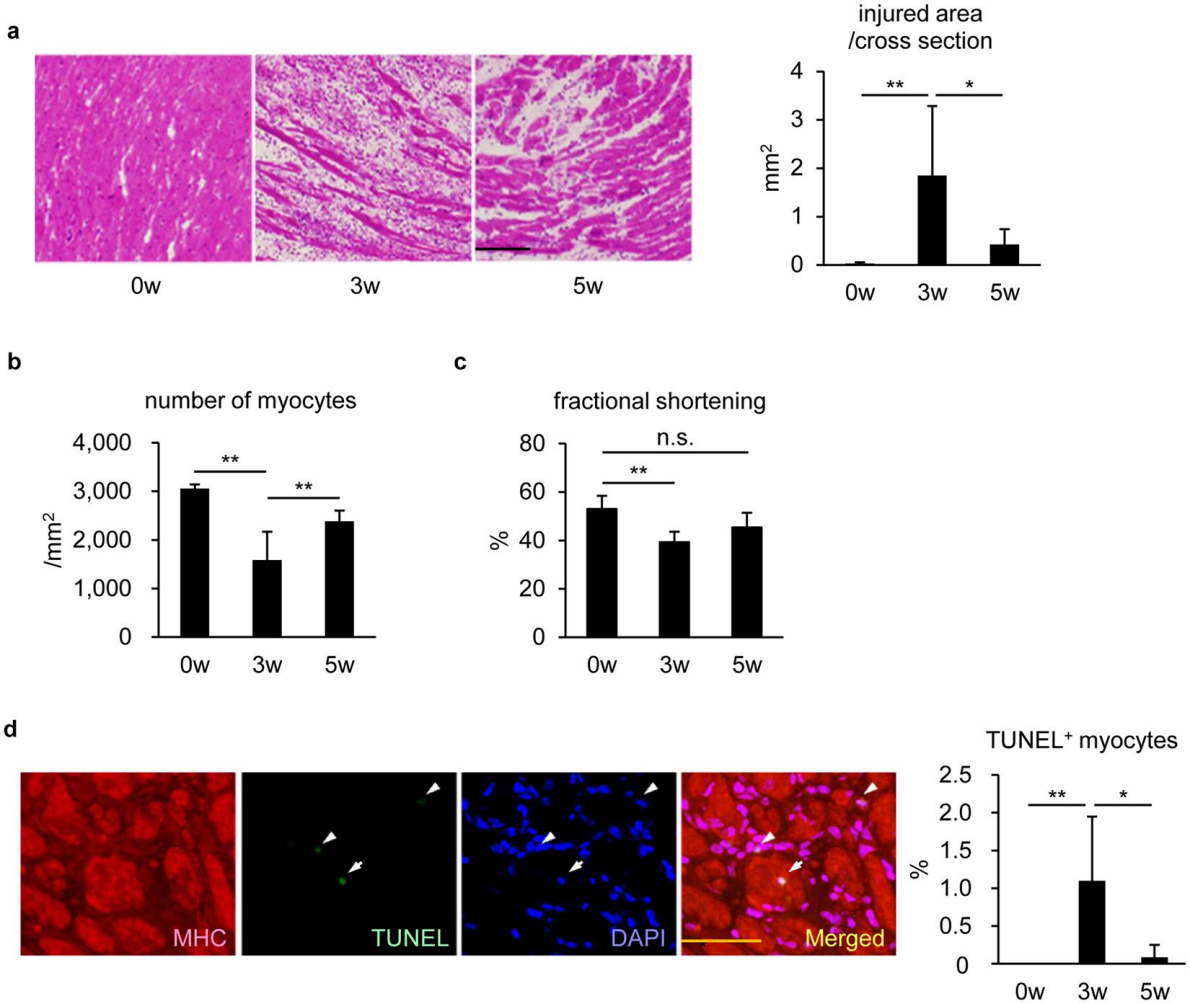
Cardiac tissue was restored from inflammation-induced injury through EAM resolution. (**a**) HE staining was performed for heart sections at the indicated time points after EAM induction. Left: representative images are shown. Scale bar: 100 μm. Right: injured area was measured. Data were from 6 mice for each group. (**b**) The number of cardiomyocytes was counted in intact, inflamed, and post-inflamed regions at EAM0w, EAM3w and EAM5w, respectively, and the density was calculated. More than 20,000 myocytes were counted from 6 mice for each group. (**c**) Fractional shortening was evaluated by echocardiography at the indicated time points after EAM induction. n = 6 mice for each group. (**d**) TUNEL staining was performed for heart sections at the indicated time points after EAM induction. Left: representative images of a TUNEL^+^MHC^+^ cell at EAM3w are shown. Arrows: a TUNEL^+^ nucleus in a MHC^+^ cell. Arrowheads: TUNEL^+^ nuclei in MHC^−^ cells. Scale bar: 50 μm. Right: TUNEL^+^MHC^+^ cells in the inflamed region were counted and shown as percentage in MHC^+^ cells. n = 5 mice for each group. (**a**,**b**,**d**) Kruskal-Wallis test; (**c**) one-way repeated measures ANOVA. *P < 0.05, **P < 0.01. Data are shown as mean ± s.d.

EAM was induced by immunization with peptides derived from mouse α-myosin heavy chain (α-MHC) twice with 7-day interval in 8 week old male BALB/c mice. Similar to human myocarditis, EAM was self-limiting; cardiac tissue was severely injured by infiltration of inflammatory cells 3 weeks after the first immunization (EAM3w). However, the damage was spontaneously attenuated at EAM5w (Fig. 1a and Supplementary Figure S1). We examined whether myocardial recovery was associated with the replenishment of cardiomyocyte density by counting the number of cardiomyocytes in intact regions before EAM induction (EAM0w), in inflamed regions at EAM3w and in post-inflamed regions at EAM5w. Cardiomyocyte density was reduced at EAM3w, followed by substantial recovery (Fig. 1b). Functionally, fractional shortening was significantly (*P* = 0.002) reduced at EAM3w but restored comparable to the basal level at EAM5w (Fig. 1c). Furthermore, the frequency of TUNEL^+^ cardiomyocytes was increased at EAM3w and diminished at EAM5w (Fig. 1d). Taken together, it is conceivable that regenerative responses were activated after EAM3w to counteract the cardiomyocyte loss.

### Cardiomyocytes positively stained with cell cycle markers were observed during EAM

**Figure 2:**
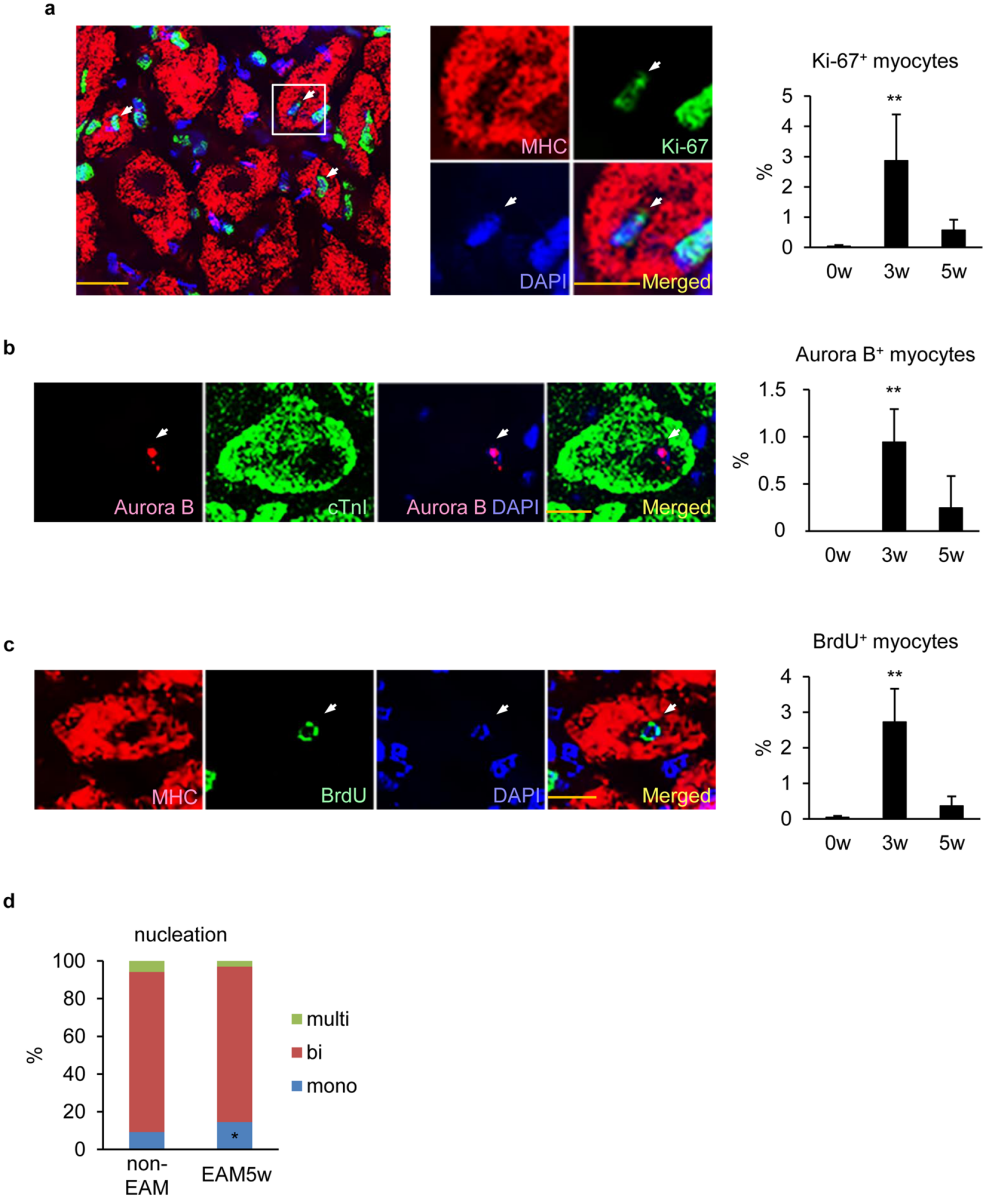
Cardiomyocytes positively stained with cell cycle markers were observed during EAM. (**a**) Heart sections were immunostained for Ki-67 and MHC at the indicated time points after EAM induction. Left: representative images of Ki-67^+^MHC^+^ cells at EAM3w are shown. Middle: magnified images of a Ki-67^+^MHC^+^ cell in the left panel. Arrows: Ki-67^+^ nuclei in MHC^+^ cells. Scale bar: 20 μm in the left panel and 10 μm in the center panel. Right: Ki-67^+^MHC^+^ cells in the inflamed region were counted and shown as percentage in MHC^+^ cells. n = 5 mice for each group. (**b**) Heart sections were immunostained for Aurora B and cTnI at the indicated time points after EAM induction. Left: representative images of an Aurora B^+^cTnI^+^ cell at EAM3w are shown. Arrows: an Aurora B^+^ nucleus in a cTnI^+^ cell. Scale bar: 10 μm. Right: Aurora B^+^cTnI^+^ cells in the inflamed region were counted and shown as percentage in cTnI^+^ cells. n = 5 mice for each group. (**c**) BrdU was intraperitoneally injected four times at the indicated time points after EAM induction. Heart sections were immunostained with anti-BrdU and anti-MHC antibodies 24 hours after the last injection. Left: representative images of a BrdU^+^MHC^+^ cell at EAM3w are shown. Arrows: a BrdU^+^ nucleus in a MHC^+^ cell. Scale bar: 10 μm. Right: BrdU^+^MHC^+^ cells in the inflamed region were counted and shown as percentage in MHC^+^ cells. n = 5 mice for 0w and 5w; 4 mice for 3w. (**d**) Cardiomyocytes were isolated from EAM5w hearts or from age-matched non-EAM hearts and stained with anti-α-actinin antibody and DAPI. A total of 1243 cardiomyocytes from 4 non-EAM hearts and 2330 cardiomyocytes from 7 EAM5w hearts were classified according to the number of nuclei. (**a**–**c**) Kruskal-Wallis test. **P < 0.01 vs 0w. (**d**) Welch’s *t*-test. *P < 0.05 vs non-EAM. Data are shown as mean ± s.d.

To examine whether cardiomyocyte proliferation compensated for the loss during myocardial restoration from EAM, cardiac sections were immunostained for cell cycle markers, Ki-67 or Aurora B, with cardiomyocyte markers, MHC or cardiac Troponin I (cTnI), respectively, and subjected to confocal microscopic observation. Similar to the previous report^25^, 0.05 ± 0.04% of cardiomyocytes were Ki-67^+^ at the baseline. The frequency of Ki-67^+^MHC^+^ cells was increased to 3.09 ± 1.24% at EAM3w, the starting point of tissue restoration, and restrained to 0.57 ± 0.35% by EAM5w (Fig. 2a). Considering that Ki-67^+^ cardiomyocytes are 0–2% after MI in adult mice^26–28^, the frequency of Ki-67^+^ myocytes was distinguishably high in EAM. Consistently, Aurora B^+^cTnI^+^ cells were increased at EAM3w (EAM0w; undetectable, EAM3w; 0.94 ± 0.35%, EAM5w; 0.25% ± 0.34%) (Fig. 2b), indicating cytokinesis of cardiomyocytes. We also analyzed the uptake of bromodeoxyuridine (BrdU) into cardiomyocytes by injecting BrdU at EAM0w, EAM3w and EAM5w and found increased frequency of BrdU^+^MHC^+^ cells in the inflamed region at EAM3w (EAM0w; 0.05 ± 0.04%, EAM3w; 2.73 ± 0.93%, EAM5w; 0.37% ± 0.27%) (Fig. 2c). Taken together, some fraction of cardiomyocytes reentered the cell cycle in response to inflammation in the healing process of EAM.

Since cardiomyocyte cell cycle progression does not necessarily result in cell division but can lead to bi/multinucleation or polyploidization^29^, we counted the nuclei of isolated cardiomyocytes at EAM5w when the myocardium was restored from injury. The proportion of mononucleated cardiomyocytes was significantly (*P* = 0.02) increased in EAM5w mice compared with the age-matched non-EAM mice (Fig. 2d). These findings are consistent with the previous reports that mononucleated cardiomyocytes preferentially proliferate compared with bi/multinucleated cells^30, 31^, suggesting that mononucleated cardiomyocytes reentered the cell cycle and underwent cell division during the resolving phase of EAM.

### Proliferative cardiomyocytes were derived from pre-existing myocytes in EAM

**Figure 3:**
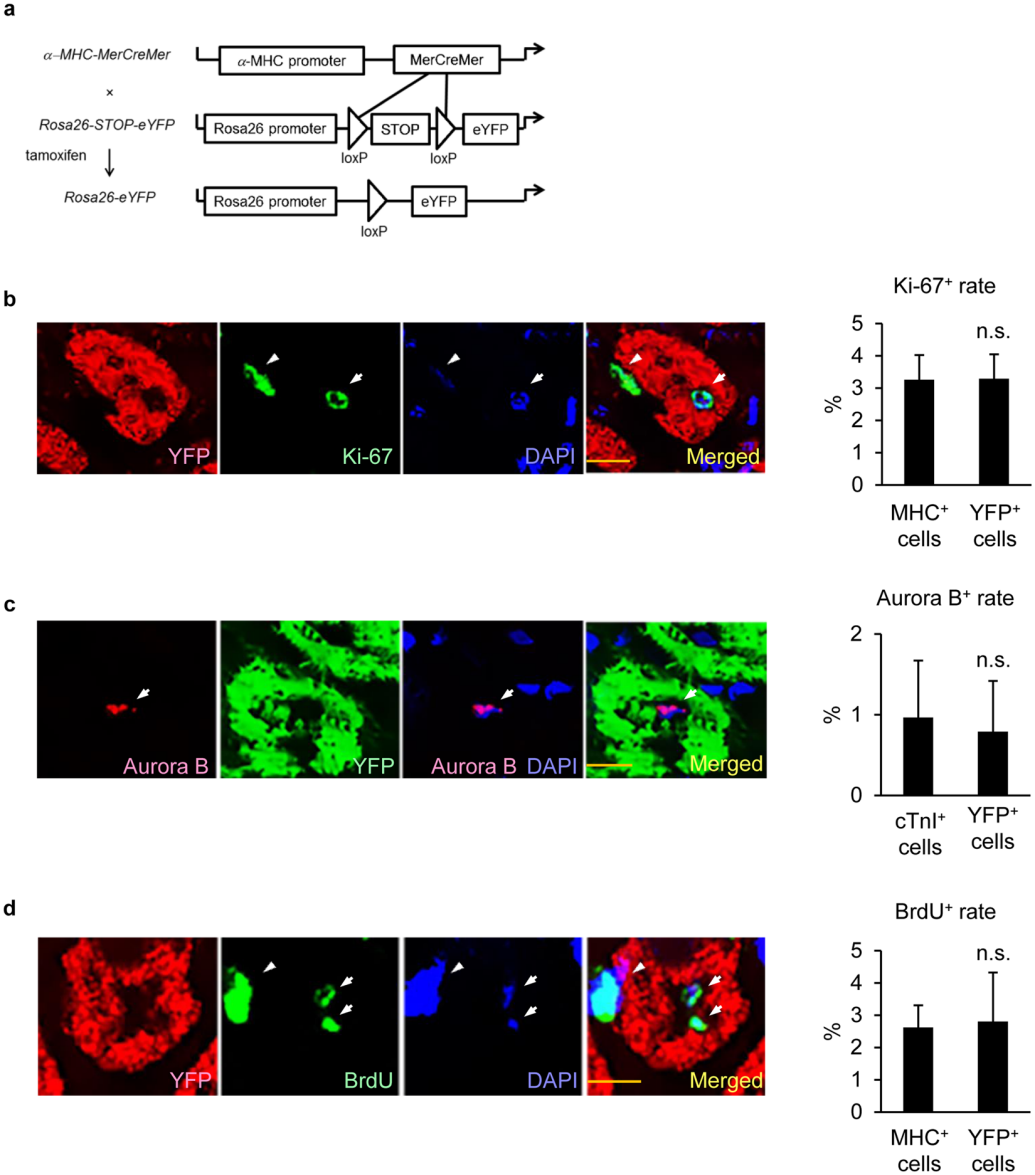
Proliferative cardiomyocytes were derived from pre-existing myocytes in EAM. (**a**) Tamoxifen was injected to double transgenic mice with α*-MHC-MerCreMer* and *Rosa26-STOP-eYFP* to label pre-existing cardiomyocytes with eYFP before EAM induction. (**b**) Heart sections were immunostained with anti-Ki-67 and anti-MHC or YFP antibodies at EAM3w. Left: representative images of a Ki-67^+^YFP^+^ cell are shown. Arrows: a Ki-67^+^ nucleus in a YFP^+^ cell. Arrowheads: a Ki-67^+^ nucleus in a YFP^−^ cell. Scale bar: 10 μm. Right: Ki-67^+^MHC^+^ and Ki-67^+^YFP^+^ cells in the inflamed region were counted and shown as percentage in MHC^+^ and in YFP^+^ cells, respectively. n = 4 mice for each group. (**c**) Heart sections were immunostained with anti-Aurora B and anti-cTnI or YFP antibodies at EAM3w. Left: representative images of an Aurora B^+^YFP^+^ cell are shown. Arrows: an Aurora B^+^ nucleus in a YFP^+^ cell. Scale bar: 10 μm. Right: Aurora B^+^cTnI^+^ and Aurora B^+^YFP^+^ cells in the inflamed region were counted and shown as percentage in cTnI^+^ and in YFP^+^ cells, respectively. n = 4 mice for each group. (**d**) BrdU was intraperitoneally injected four times at EAM3w. Heart sections were immunostained with anti-BrdU and anti-MHC or YFP antibodies 24 hours after the last injection. Left: representative images of a BrdU^+^YFP^+^ cell are shown. Arrows: BrdU^+^ nuclei in a YFP^+^ cell. Arrowheads: a BrdU^+^ nucles in a YFP^−^ cell. Scale bar: 10 μm. Right: BrdU^+^MHC^+^ and BrdU^+^YFP^+^ cells in the inflamed region were counted and shown as percentage in MHC^+^ and in YFP^+^ cells, respectively. n = 4 mice for each group. (**b**–**d**) Welch’s *t*-test. Data are shown as mean ± s.d.

To make clear whether proliferative cardiomyocytes were derived from pre-existing myocytes, we performed cell fate mapping analyses. We established transgenic mice which express eYFP in cardiomyocytes in a tamoxifen-inducible manner^32^ (Fig. 3a). Pre-existing cardiomyocytes were specifically labeled with eYFP by tamoxifen injection, followed by EAM induction. Before conducting cell cycle analyses at EAM3w, we confirmed that ~80% of cardiomyocytes were labeled with eYFP at this point (Supplementary Figure S2) as previously reported^33, 34^. Ki-67 expression analysis demonstrated that the frequency of Ki-67^+^YFP^+^ cells in EAM3w hearts was equivalent to that of Ki-67^+^MHC^+^ cells in the same hearts (Fig. 3b). Similar results were obtained from Aurora B staining and BrdU incorporation assays (Fig. 3c,d), indicating that pre-existing cardiomyocytes, rather than non-cardiomyocyte population, such as precursors and stem cells, primarily contributed to cardiomyocyte proliferation, as is the common case with cardiac regeneration observed in zebrafish and neonatal murine hearts after injury^13–15, 17^. These results suggest that adult mammalian cardiomyocytes at least partially restored cell cycle activities during myocarditis.

### STAT3 was activated in cardiomyocytes at EAM3w

**Figure 4:**
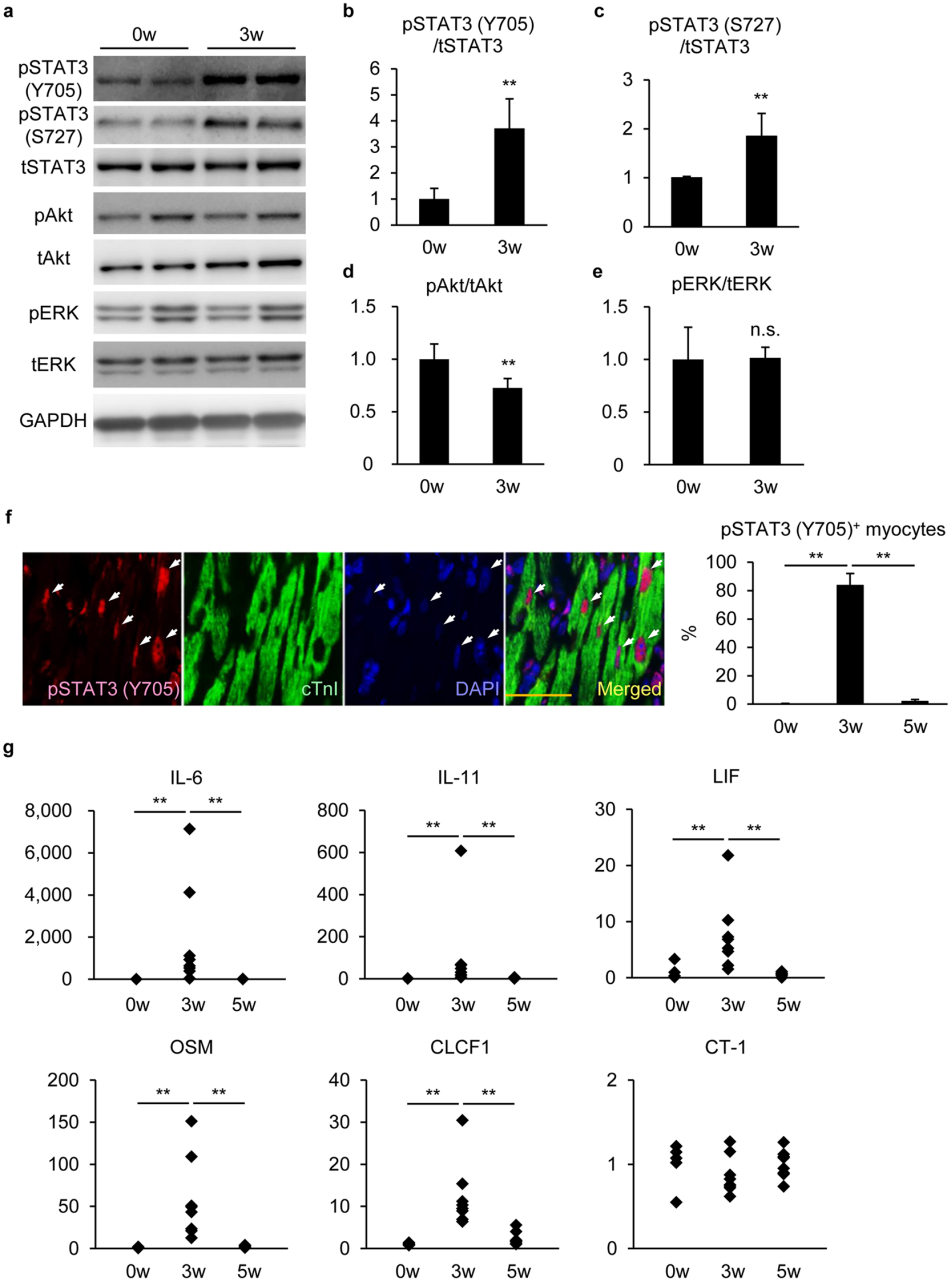
STAT3 was activated in cardiomyocytes at EAM3w. (**a**) Heart homogenates at EAM0w and EAM3w were subjected to immunoblotting with anti-phosphorylated STAT3 (pSTAT3) (Y705), anti-pSTAT3 (S727), anti-pAkt, anti-pERK and anti-GAPDH antibodies. Blots were reprobed with anti-total STAT3 (tSTAT3), anti-tAkt and anti-tERK antibodies. The full-length blots are presented in Supplementary Figure S3. (**b**–**e**) The band intensity was measured with ImageJ and normalized to that of GAPDH. Data are shown as fold increase relative to 0w. n = 4 mice for 0w; 6 mice for 3w. (**f**) Heart sections were immunostained with anti-pSTAT3 (Y705) and anti-cTnI antibodies at the indicated time points after EAM induction. Left: representative images of pSTAT3 (Y705)^+^cTnI^+^ cells at EAM3w are shown. Arrows: pSTAT3 (Y705)^+^ nuclei in cTnI^+^ cells. Scale bar: 50 μm. Right: pSTAT3 (Y705)^+^cTnI^+^ cells in the inflamed region were counted and shown as percentage in cTnI^+^ cells. n = 3 mice for each group. (**g**) The expression of IL-6, IL-11, LIF, OSM, CLCF1 and CT-1 transcripts was quantified at EAM0w and EAM3w by quantitative RT-PCR. The expression of these genes was normalized to that of gapdh and shown as fold increase relative to 0w. n = 5 mice for 0w; 8 mice for 3w and 5w. (**b**–**e**) Welch’s *t*-test; (**f**) Kruskal-Wallis test; (**g**) Steel-Dwass test. **P < 0.01. Data are shown as mean ± s.d. in (**b**–**f**).

Several signaling pathways, including STAT3, Akt and ERK, have been proposed to be cardioprotective and proliferative factors in zebrafish and neonatal mice^5, 11, 12, 35–39^. Therefore, to explore the signaling pathways that drive cardiomyocyte proliferation under myocarditis, we examined whether these signaling pathways were activated during EAM. Immunoblot analyses showed that phosphorylation of STAT3 at Y705, the essential residue for the transcriptional activity^40^, was enhanced at EAM3w. Additionally, phosphorylation of STAT3 at S727 which is required for boosting the transcriptional activity as well as non-canonical activities, such as mitochondrial protection^40^, was also enhanced, while Akt or ERK was not activated at this point (Fig. 4a–e and Supplementary Figure S3). Immunostaining with anti-pSTAT3 (Y705) antibody revealed that STAT3 was translocated to cardiomyocyte nuclei in the inflamed region at EAM3w, which was almost reverted at EAM5w (EAM0w; 0.3 ± 0.2%, EAM3w; 83.6 ± 8.4%, EAM5w; 1.9 ± 1.4%) (Fig. 4f). The attenuated activation of STAT3 at EAM5w was also demonstrated by immunoblotting (Supplementary Figure S4).

Since STAT3 is known to be activated by IL-6 family cytokine stimulation^6^, the mRNA expression of IL-6 family cytokines was examined. A variety of IL-6 family cytokines, i.e., IL-6, IL-11, leukemia inhibitory factor (LIF), oncostatin M (OSM) and cardiotrophin-like cytokine factor 1 (CLCF1) were remarkably upregulated at EAM3w, while cardiotrophin-1 (CT-1) was not. In accordance with STAT3 activity, the upregulation of IL-6 family cytokines was attenuated by EAM5w (Fig. 4g).

### *STAT3* gene ablation suppressed the frequency of proliferative cardiomyocytes with impaired myocardial restoration from EAM

**Figure 5:**
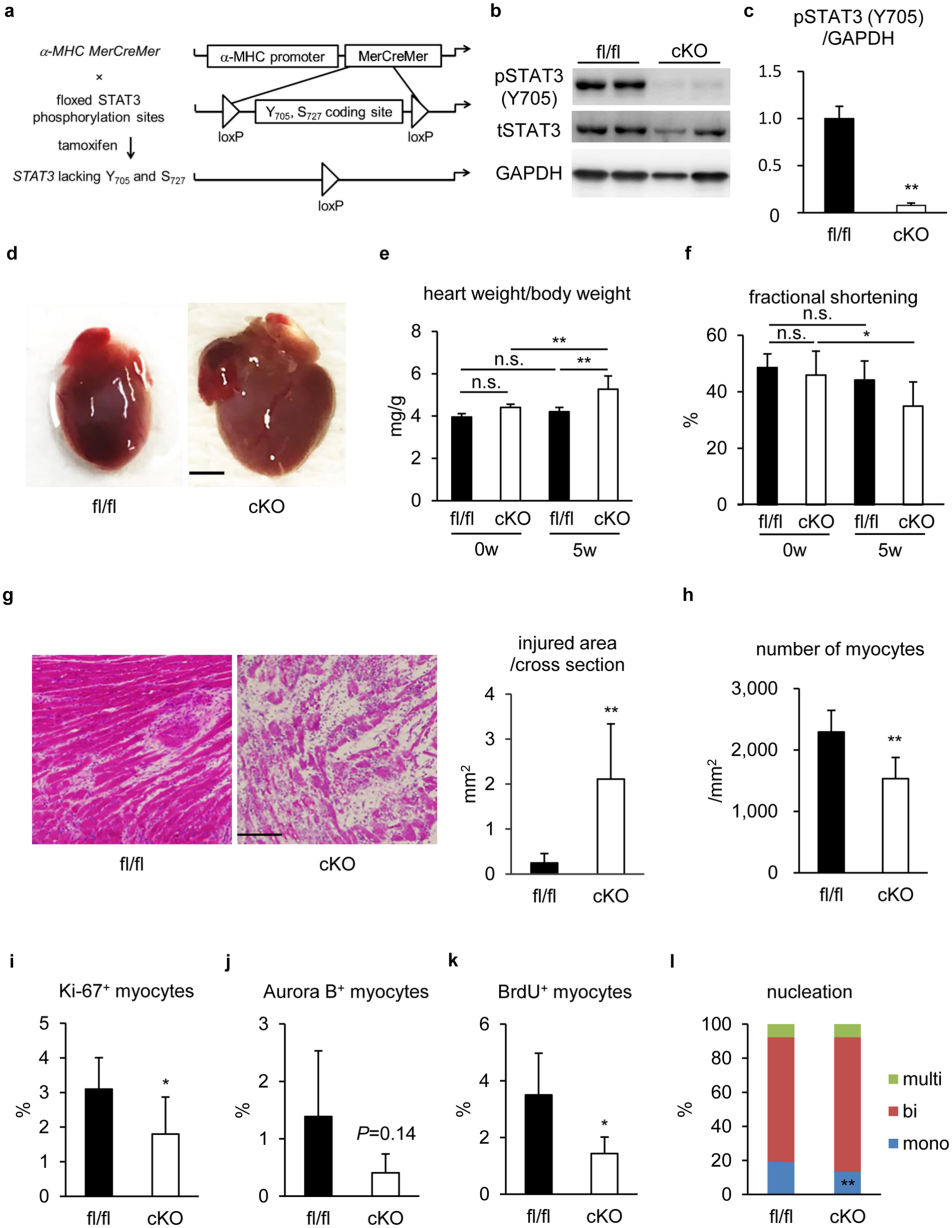
*STAT3* gene ablation suppressed the frequency of proliferative cardiomyocytes with impaired myocardial restoration from EAM. (**a**) Tamoxifen was injected to double transgenic mice with α*-MHC-MerCreMer* and *STAT3* flox to ablate *STAT3* gene in cardiomyocytes before EAM induction. (**b**) Cardiomyocytes isolated from STAT3fl/fl and STAT3cKO hearts at EAM3w were subjected to immunoblotting with anti-pSTAT3 (Y705), anti-tSTAT3 and anti-GAPDH antibodies. The full-length blots are presented in Supplementary Figure S5. (**c**) The band intensity was measured with ImageJ and normalized to that of GAPDH. Data are shown as fold increase relative to fl/fl. n = 7 mice for fl/fl; 4 mice for cKO. (**d**) Representative STAT3fl/fl and STAT3cKO hearts at EAM5w. Scale bar: 2 mm. (**e**) The ratio of heart weight to body weight was calculated for the indicated groups. n = 5 mice for fl/fl 0w and cKO 0w; 7 mice for fl/fl 5w; 14 mice for cKO 5w. (**f**) Fractional shortening of STAT3fl/fl and STAT3cKO mice was evaluated by echocardiography at EAM0w and EAM5w. n = 9 mice for each group. (**g**) HE staining was performed for heart sections from STAT3fl/fl and STAT3cKO mice at EAM5w. Left: representative images are shown. Scale bar: 100 μm. Right: injured area was measured. Data were from 7 mice for each group. (**h**) The number of cardiomyocytes in post-inflamed areas of STAT3fl/fl and STAT3cKO hearts was counted at EAM5w and the density was calculated. More than 18,000 myocytes were counted from 7 mice for each group. (**i**) Heart sections from STAT3fl/fl and STAT3cKO mice at EAM3w were immunostained for Ki-67 and MHC. Ki-67^+^MHC^+^ cells in the inflamed region were counted and shown as percentage in MHC^+^ cells. n = 5 mice for fl/fl; 8 mice for cKO. (**j**) Heart sections from STAT3fl/fl and STAT3cKO mice at EAM3w were immunostained for Aurora B and cTnI. Aurora B^+^cTnI^+^ cells in the inflamed region were counted and shown as percentage in cTnI^+^ cells. n = 5 mice for fl/fl; 8 mice for cKO. (**k**) BrdU was intraperitoneally injected four times into STAT3fl/fl and STAT3cKO mice at EAM3w. Heart sections were immunostained for BrdU and MHC 24 hours after the last injection. BrdU^+^MHC^+^ cells in the inflamed region were counted and shown as percentage in MHC^+^ cells. n = 5 mice for fl/fl; 8 mice for cKO. (**l**) Cardiomyocytes were isolated from STAT3fl/fl or STAT3cKO mice at EAM5w and stained with anti-α-actinin antibody and DAPI. A total of 1296 cardiomyocytes from 5 STAT3fl/fl hearts and 1367 cardiomyocytes from 5 STAT3cKO hearts were classified according to the number of nuclei. (**c**,**g**–**l**) Welch’s *t*-test; (**e**) two-way factorial ANOVA; (**f**) two-way repeated measures ANOVA. *P < 0.05, **P < 0.01. Data are shown as mean ± s.d.

To determine the pathophysiological roles of STAT3 in EAM, we established cardiomyocyte-specific STAT3 conditional knockout (STAT3cKO) mice using the tamoxifen-inducible system^32^. In this system, the phosphorylation region of STAT3 molecule is selectively deleted^41^ (Fig. 5a). We administered tamoxifen in advance to EAM induction, and diminished pSTAT3 (Y705) expression was confirmed by immunoblotting of isolated cardiomyocytes at EAM3w (Fig. 5b,c and Supplementary Figure S5). EAM was induced in STAT3cKO mice and STAT3fl/fl mice without *MerCreMer* transgene, control mice, and histological and functional analyses were performed at EAM5w. At this point, STAT3cKO hearts were visually larger in size than STAT3fl/fl hearts (Fig. 5d), which was supported by increased heart weight (HW) to body weight (BW) ratio (Fig. 5e). Echocardiographic analyses revealed that fractional shortening was reduced at EAM5w compared to EAM0w in STAT3cKO mice, while not in STAT3fl/fl mice that had recovered by EAM5w (Fig. 5f and Supplementary Table S1). Moreover, histological analyses demonstrated that tissue restoration was impaired in STAT3cKO hearts (Fig. 5g and Supplementary Figure S6), which was associated with decreased cardiomyocyte density in the post-inflamed region (Fig. 5h). There was no significant difference in capillary density between STAT3fl/fl and STAT3cKO hearts at EAM5w (Supplementary Figure S7), indicating that endothelial cells were minimally affected by cardiomyocyte-specific deletion of *STAT3* gene.

It is widely accepted that STAT3 plays important roles in cardioprotection through activating transcription of cardioprotective molecules^5^. Therefore, we examined cardiomyocyte apoptosis at EAM3w and found that TUNEL^+^ cardiomyocytes were increased in STAT3cKO mice (fl/fl; 1.38 ± 0.12%, cKO; 2.24 ± 0.38%) (Supplementary Figure S8).

Next, to investigate the effect of STAT3 deletion on cardiomyocyte proliferation, we examined the expression of cell cycle markers at EAM3w. Importantly, the frequency of Ki-67^+^ cardiomyocytes in the inflamed region was reduced in STAT3cKO mice (fl/fl; 3.10 ± 0.91%, cKO; 1.80 ± 1.07%) (Fig. 5i). Though not statistically significant (*P* = 0.14), Aurora B^+^ cardiomyocytes seemed to be also decreased in STAT3cKO mice (fl/fl; 1.39 ± 1.14%, cKO; 0.41 ± 0.33%) (Fig. 5j). Furthermore, cardiomyocyte BrdU incorporation was decreased in STAT3cKO mice (fl/fl; 3.50 ± 1.47%, cKO; 1.44 ± 0.58%) (Fig. 5k). Consistently, the proportion of mononucleated cardiomyocytes was significantly (*P* = 0.004) reduced in STAT3cKO hearts at EAM5w (Fig. 5l).

We also examined whether STAT3 stimulation could enhance cardiomyocyte proliferation at EAM3w by intravenously administering IL-11, which activates cardiomyocyte STAT3, as reported previously^8, 9^. As a result, IL-11-treated mice showed increased population of Ki-67^+^ or Aurora B^+^ cardiomyocytes compared to PBS-treated mice (Supplementary Figure S9a,b). Similarly, BrdU^+^ cardiomyocytes tended to be slightly increased by IL-11 administration (Supplementary Figure S9c). These results propose STAT3 activation as a promising strategy for cardiac regeneration at least when cardiomyocytes are predisposed to proliferate.

### Fibrosis was augmented in STAT3cKO hearts at EAM5w

**Figure 6:**
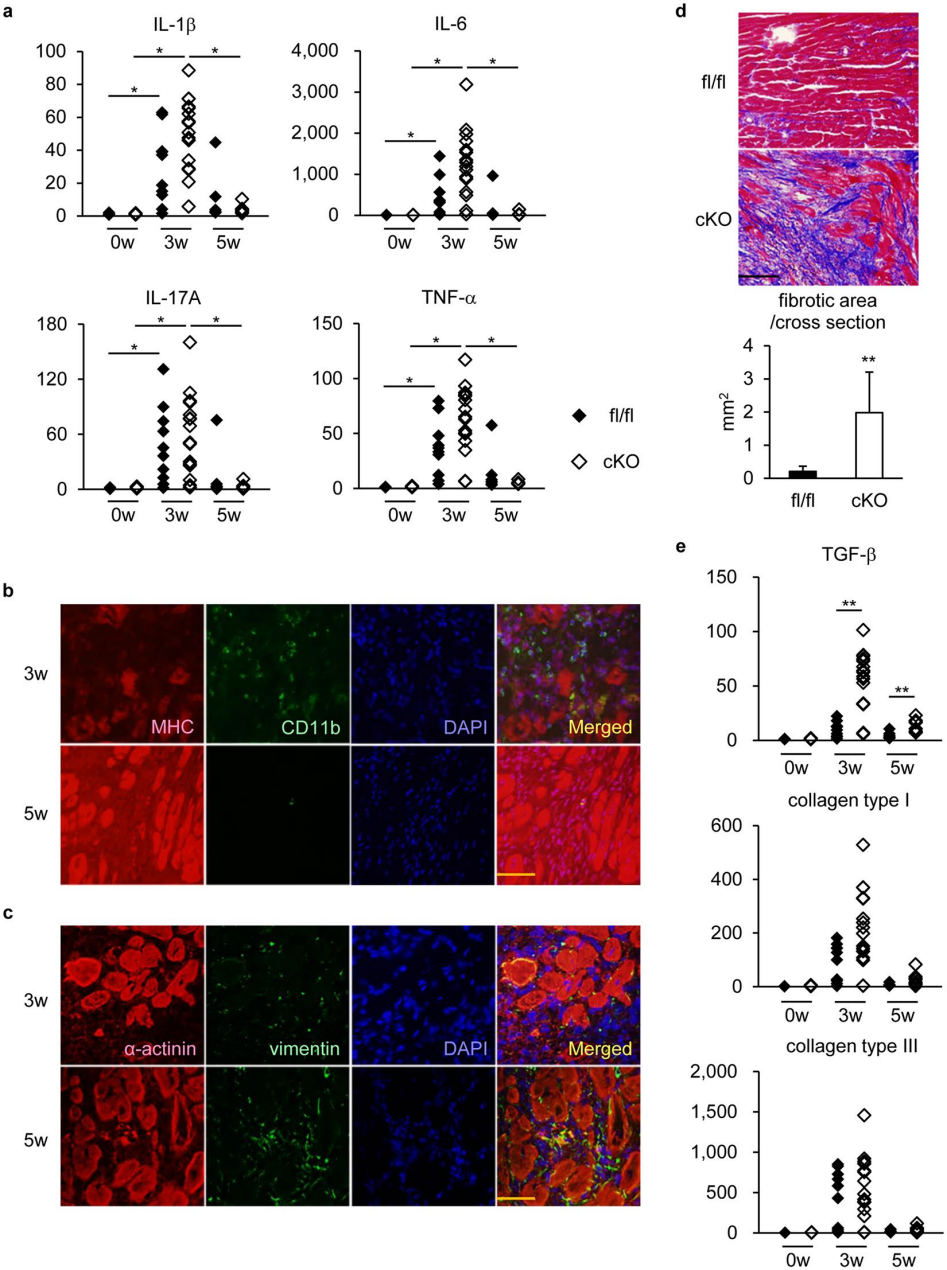
Fibrosis was augmented in STAT3cKO hearts at EAM5w. (**a**) The expression of IL-1β, IL-6, IL-17A and TNF-α transcripts in STAT3fl/fl and STAT3cKO hearts was quantified at the indicated time points after EAM induction by quantitative RT-PCR. The expression of these genes was normalized to that of gapdh and shown as fold increase relative to fl/fl 0w. n = 5 mice for fl/fl 0w and cKO 0w; 10 mice for fl/fl 3w; 16 mice for cKO 3w; 7 mice for fl/fl 5w; 8 mice for cKO 5w. (**b**) Heart sections from STAT3cKO mice at EAM3w and EAM5w were immunostained for MHC and CD11b. Scale bar: 50 μm. (**c**) Heart sections from STAT3cKO mice at EAM3w and EAM5w were immunostained for α-actinin and vimentin. Scale bar: 50 μm. (**d**) Masson’s trichrome staining was performed for heart sections from STAT3fl/fl and STAT3cKO mice at EAM5w. Top: representative images are shown. Scale bar: 100 μm. Bottom: fibrotic area was measured. Data were from 7 mice for each group. (**e**) The expression of TGF-β, collagen type I and collagen type III transcripts in STAT3fl/fl and STAT3cKO hearts was quantified at the indicated time points after EAM induction by quantitative RT-PCR. The expression of these genes was normalized to that of gapdh and shown as fold increase relative to fl/fl 0w. n = 5 mice for fl/fl 0w and cKO 0w; 10 mice for fl/fl 3w; 16 mice for cKO 3w; 7 mice for fl/fl 5w; 8 mice for cKO 5w. (**a**,**e**) Steel-Dwass test; (**d**) Welch’s *t*-test. *P < 0.05, **P < 0.01. Data are shown as mean ± s.d. in (**d**).

We analyzed the pathological feature of the impaired recovery of STAT3cKO hearts from EAM-induced injury. First, in order to examine whether cardiomyocyte *STAT3* gene deletion led to sustained myocardial inflammation, the severity of inflammation was evaluated by measuring mRNA expression of inflammatory cytokines. The expression of inflammatory cytokines, IL-1β, IL-6, IL-17A and TNF-α, was upregulated with the peak at EAM3w and, afterwards, declined near to the basal level at EAM5w in both STAT3fl/fl and STAT3cKO hearts (Fig. 6a), indicating that cardiomyocyte STAT3 deletion did not elongate inflammation. Consistently, although a majority of injured area in STAT3cKO hearts was occupied by CD11b^+^ myeloid cells at EAM3w as described previously^42^, they were replaced prominently with vimentin^+^ fibroblasts at EAM5w (Fig. 6b,c). Masson’s trichrome staining revealed abundant and rigid collagen fiber formation in the injured regions of STAT3cKO hearts at EAM5w (Fig. 6d), which was confirmed by picrosirius red staining (Supplementary Figure S10). Since fibrotic area was comparable to the injured area that was not occupied by cardiomyocytes, fibrosis could be the dominant alternative way to fill the regions where cardiomyocytes failed to propagate, as is the case in MI^3, 4^. Consistently, the expression of TGF-β, the key fibrotic factor^43^, increased at EAM3w and EAM5w in STAT3cKO hearts (Fig. 6e). Similarly, the expression of collagen type I transcript showed a tendency to increase in STAT3cKO hearts, although no difference was observed in the expression of collagen type III between STAT3cKO and STAT3fl/fl hearts (Fig. 6e). These data suggest that the impaired tissue repair/regeneration in STAT3cKO hearts was due to increased death and decreased proliferation of cardiomyocytes accompanied by excessive fibrosis, rather than prolonged inflammation.

### The expression of regeneration-related genes, *metallothioneins* and *clusterin*, was downregulated in STAT3cKO cardiomyocytes

**Figure 7:**
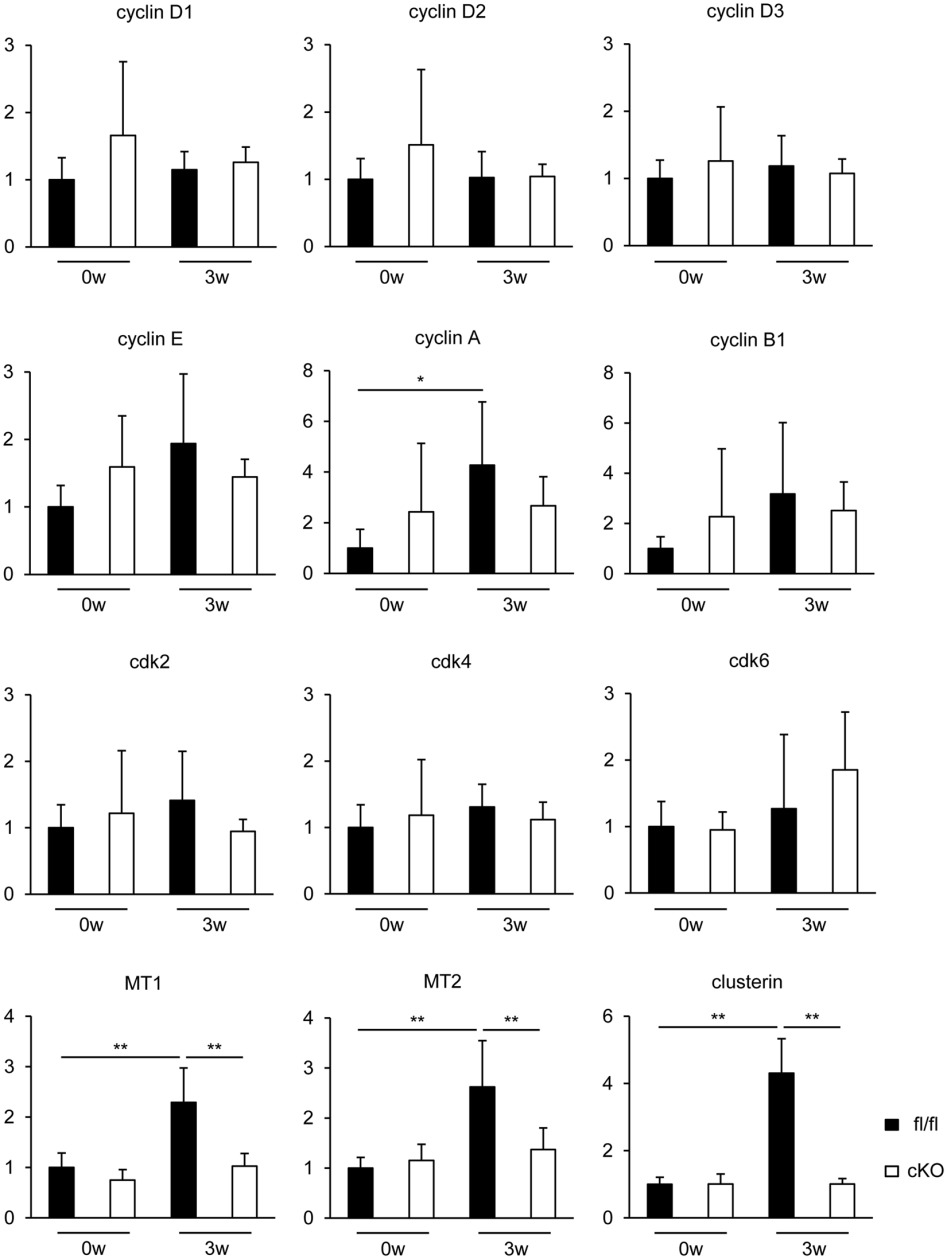
The expression of regeneration-related genes, *metallothioneins* and *clusterin*, was downregulated in STAT3cKO cardiomyocytes. Cardiomyocytes were isolated from STAT3fl/fl and STAT3cKO hearts at EAM0w and EAM3w. Cyclin D1, cyclin D2, cyclin D3, cyclin E, cyclin A, cyclin B1, cdk2, cdk4, cdk6, MT1, MT2 and clusterin mRNA expression was quantified by quantitative RT-PCR. The expression of these genes was normalized to that of gapdh and shown as fold increase relative to fl/fl 0w. n = 6 mice for fl/fl 0w and cKO 0w; 8 mice for fl/fl 3w and cKO 3w. Two-way factorial ANOVA. *P < 0.05, **P < 0.01. Data are shown as mean ± s.d.

In order to address the mechanistic aspects underlying the impaired tissue repair/regeneration of STAT3cKO hearts, microarray analysis was performed to analyze the difference in the transcriptome between STAT3fl/fl and STAT3cKO cardiomyocytes at EAM3w (Supplementary Dataset 1). Drastic changes were not observed in the expression of cell cycle regulators, such as cyclins, supposedly due to the limited frequency of cardiomyocytes reentering the cell cycle. The expression of cyclins and cdks, which are particularly important for embryonic heart development^44^, was also examined by quantitative RT-PCR, confirming the comparable expression between STAT3fl/fl and STAT3cKO cardiomyocytes, although cyclin A expression at EAM3w was increased only in STAT3fl/fl cardiomyocytes when compared to EAM0w (Fig. 7). In EAM model, the frequency of proliferating or Ki-67^+^ cardiomyocytes was about 3% in the inflamed area, which was presumably not sufficient to strongly affect the expression value of cyclins or cdks with the masking effect of quiescent cardiomyocytes. On the other hand, microarray analysis revealed downregulation of other potential regeneration-related genes, *metallothionein 1* (*MT1*), *MT2* and *clusterin*, in STAT3cKO cardiomyocytes. In spite of the increased expression of TGF-β in STAT3cKO whole hearts, there was no difference in TGF-β expression between STAT3fl/fl and STAT3cKO cardiomyocytes, indicating that TGF-β is produced from non-cardiomyocyte population under the control of interaction between cardiomyocytes and non-cardiomyocytes.

Quantitative RT-PCR verified the decreased expression of MT1 and MT2 in STAT3cKO cardiomyocytes (Fig. 7). We have previously shown that MT1 and MT2 are targets of STAT3 and exhibit cardioprotective functions as reactive oxygen species (ROS) scavengers^9^. Therefore, the reduced expression of MT1 and MT2 might result in the increased cardiomyocyte apoptosis in STAT3cKO hearts with decreased resistance against ROS. In addition to cytoprotection, it is also reported that the expression of MTs is positively associated with cell proliferation particularly in malignancies^45–48^, possibly though the maintenance of zinc homeostasis^49^.

We also found that the expression of clusterin was downregulated in STAT3cKO cardiomyocytes at EAM3w (Fig. 7). Clusterin is known to be a downstream factor of IL-11^50^ and to exert multifunctionality in a variety of circumstances including tumorigenesis^51, 52^. Importantly, clusterin promotes tumor progression by accelerating cell proliferation, although the precise underlying mechanisms remain to be fully elucidated^52^. Of note, clusterin is required for restricting myocarditis-induced damage^53, 54^. Clusterin-deficient mice exhibit severer myocardial scarring and functional impairment after autoimmune myocarditis^54^.

## Discussion

It is generally accepted that adult mammalian cardiomyocytes are postmitotic and that their proliferation contributes to tissue repair limitedly, if any, after myocardial damage^2^. Here, in the murine EAM model, we demonstrated that myocardial damage induced by myocarditis was attenuated in the healing process, accompanied by cell cycle reentry of adult cardiomyocytes. The source of cycling cardiomyocytes was found to be pre-existing cardiomyocytes rather than non-cardiomyocyte population, i.e., precursors/stem cells. Importantly, STAT3 was robustly activated during inflammation, and STAT3cKO hearts exhibited impaired recovery from inflammatory damage due to increased apoptosis and decreased proliferation of cardiomyocytes. Additionally, IL-11 treatment increased the cardiomyocytes that were positively stained for cell cycle markers. Microarray analysis revealed decreased expression of regeneration-related genes, such as *MT1*, *MT2* and *clusterin*, in STAT3-deficient cardiomyocytes. Collectively, these results indicate that STAT3 acts not only as a protective factor but also as a proliferative factor in adult mammalian cardiomyocytes, contributing to cardiac repair/regeneration from myocarditis-induced damage.

In this study, we evaluated cell cycle reentry of cardiomyocytes based on several markers, in line with the previous studies^26–28, 31, 55^. One of the most important findings is that a significant portion of cardiomyocytes expressed Aurora B, a marker of mitotic phase, during myocarditis. The frequency of Aurora B^+^ cardiomyocytes in EAM was remarkably high compared to that reported in MI: 0.94% in EAM hearts and less than 0.01% in infarcted hearts^31, 55^. The high Aurora B^+^ rate in EAM might contradict with the previous reports which describe the recalcitrance of adult cardiomyocytes to cytokinesis^56^; however, the ratio of Aurora B^+^ cells to Ki-67^+^ cells (0.94: 3.09) is found to be reasonable in comparison with other actively cycling tissues, considering that the ratio of Aurora B^+^ cells to Ki-67^+^ cells is similar among various types of cells, while the rate of Ki-67^+^ cells is dependent on cell types. In human oral squamous cells, for example, 4.4% of the nuclei are Aurora B^+^ and 13.7% are Ki-67^+ ^^57^.

Consistent with high rate of Aurora B^+^ myocytes in EAM, mononucleated cardiomyocytes significantly increased in frequency during EAM. Previous studies demonstrated that cardiomyocytes display low DNA synthetic activities during aging and ischemic insults^1, 26, 27^, but that bi/multinucleation and/or polyploidization precede cardiomyocyte division in these cases^34, 58^. Interestingly, experiments using genetically modified mice have demonstrated that increased population of mononucleated cardiomyocytes is associated with cardiac regenerative capacity^59, 60^. For example, meis1, a homeodomain transcription factor, was identified as a suppressor of cardiomyocyte proliferation, and its overexpression hampers neonatal cardiac regeneration, while meis1 knockout mice exhibit increased mononucleated cardiomyocytes with enhanced regenerative capacity^59^. Thus, increased cytokinesis of cardiomyocytes is a distinct feature of myocarditis from other pathophysiological conditions, such as MI, and may be indicative of potent regenerative activities of the heart.

In zebrafish, adult cardiomyocytes reenter the cell cycle and contribute to cardiac regeneration through STAT3 activation in injured myocardium^11, 13, 14^. However, there has been little evidence that adult mammalian cardiomyocyte proliferation is involved in wound healing. Here, we showed that STAT3 is activated in the inflamed heart and promotes cell cycle reentry of adult mammalian cardiomyocytes. Although STAT3 is required for cardiac tissue repair, as is the case with zebrafish^11^, activation of STAT3 signal by itself is not sufficient for restoring proliferative capacity of cardiomyocytes in adult mammals. Indeed, there was a remarkable difference in frequency between pSTAT3 positive cells and proliferative marker positive cells in EAM myocardium, indicating that additional signals are involved in the regulation of adult cardiomyocyte proliferation. In this context, it is interesting that STAT3 is also activated in infarcted hearts and mediates cardioprotection^8, 18^, but cardiomyocyte proliferation remains at a substantially lower rate in MI than that in EAM^26–28, 55^. The difference between these two models might be explained by different expression levels of positive and/or negative regulators of cell proliferation. For instance, meis1, a negative regulator of proliferation, is reported to be upregulated in MI model^59^, but we found no alteration in meis1 expression during EAM (Supplementary Figure S11). Thus, signals which are specifically activated or inactivated by EAM, but not by MI, might constitute the reparative properties of myocarditis, as inflammation-regeneration linkage. STAT3 activation by IL-11, in combination with such signals, has potential implications for cardiac regenerative therapy.

By using microarray analyses, we found that the expression of regeneration-related genes, such as *MT1*, *MT2*, and *clusterin*, was downregulated in STAT3cKO cardiomyocytes, indicating that these molecules, at least partially, contribute to cardiac regeneration in EAM as mechanistic aspects. Of note, STAT3 was phosphorylated at both Y705 and S727 during EAM, proposing the possibility that STAT3 functions not only as a transcriptional factor but as a non-transcriptional, mitochondrial regulator^61^. Given the limitation of microarray analyses, further studies might be required to understand the importance of STAT3 as a mitochondrial regulator in cardiac regeneration. Since ERK, a well-known kinase capable of phosphorylating STAT3 at S727, was not activated during EAM, other serine/threonine kinases, such as protein kinase Cε (PKCε), PKCδ, ZIP kinase, mTOR, and CDK5, may be important as upstream regulators in this respect^40^.

Recently, we have reported that cytoskeletal proteins are upregulated in cardiomyocytes and that cardiomyocytes actively form protrusions, a morphological feature of myocardial regeneration^62^, in the resolution phase of EAM^63^. The most remarkably upregulated cytoskeletal protein, moesin, potently promoted the protrusion formation of cardiomyocytes, and the protrusions mediated cardiomyocyte cell-cell contact. Therefore, cardiomyocytes are structurally modulated during EAM, leading to replenishment of cellular connections. In the present study, we examined whether cardiomyocyte loss is compensated quantitatively in EAM and demonstrated that a significant population of adult mammalian cardiomyocytes restored proliferative activities in the resolution phase. These findings would provide novel insights into the clinical features of myocarditis that define it as a self-limiting disease. Further understanding of the regulatory mechanisms of cardiomyocyte cell cycle reentry during myocarditis could pave a new way to cardiac regenerative therapy that takes advantage of intrinsic regenerative capacity of adult hearts.

## Methods

### Animal experiments

Animal care was conducted under the supervision of Animal Experimentation Committee of Osaka University in compliance with the Osaka University animal care guideline. All experimental procedures conformed to the Guide for the Care and Use of Laboratory Animals Eighth Edition updated by the US National Research Council Committee in 2011 and were approved by Animal Experimentation Committee of Osaka University.

### Experimental autoimmune myocarditis

EAM was induced as described previously^23, 24^. In brief, 8 week old male mice were immunized with cardiac-specific peptides (α-MHC_614–629_; Ac-SLKLMATLFSTYASAD-OH) twice with 7 days of interval. The peptides were diluted in PBS and emulsified with complete Freund’s adjuvant. EAM0w mice were randomly chosen from pre-immunized mice.

### Histopathology

Hearts were excised from euthanized mice and rapidly frozen in O.C.T. Compound (Sakura Finetek Japan). The frozen blocks were sliced into 5 μm-thick sections using Leica CA 1850 (Leica). After fixation with 4% paraformaldehyde (PFA) for 15 min, the sections were stained with hematoxylin and eosin (HE) or Masson’s trichrome method. Images were taken under the bright-field mode of FSX-100 (Olympus). The inflamed area, the number of cardiomyocytes and the fibrotic area were measured using ImageJ software by a researcher who was blinded to the experimental condition. Cardiomyocyte density was calculated by dividing the number of cardiomyocytes by the area in intact, inflamed and post-inflamed regions for EAM0w, EAM3w and EAM5w, respectively.

### TUNEL staining

TUNEL staining was performed using *In Situ* Apoptosis Detection Kit (Takara) according to manufacturer’s protocol with minor modifications. In brief, after frozen heart sections were fixed with 4% PFA and blocked with 3% bovine serum albumin (BSA), DNA nicks were labeled with *In Situ* Apoptosis Detection Kit. Anti-MHC primary antibody at 1/400, Alexa Fluor 546-conjugated secondary antibody at 1/400 and DAPI were applied for further staining.

### Immunofluorescence staining

Frozen heart sections were fixed with 4% PFA for 15 min and blocked with 3% BSA. Primary antibodies and Alexa Fluor 488- or 546-conjugated secondary antibodies were mounted at 1/400 on the sections in sequence. Nuclei were stained with DAPI. For cardiomyocyte cell cycle analyses, confocal observations were conducted using Leica TCS SP5 (Leica). Antibodies used are listed in Supplementary Table S2.

### BrdU incorporation assay

BrdU (1.875 mg/body) in 100 μL PBS was injected intraperitoneally four times with 2-hour intervals. Twenty-four hours after the last injection, the mice were euthanized and frozen heart sections were processed through fixation and blocking. After allowing the sections to react with anti-MHC primary antibody at 1/400, BrdU was predisposed by incubation with DNase I recombinant, RNase-free (Roche) according to manufacturer’s protocol. Anti-BrdU primary antibody at 1/400 was subsequently applied. Alexa Fluor 488- or 546-conjugated antibodies at 1/400 were used as secondary antibodies. Nuclei were stained with DAPI.

### Cardiomyocyte isolation from adult mice

Mice were euthanized 15 min after intraperitoneal heparin injection (67 U/body). The hearts were excised and instantly cannulated. Following 3 min of retrograde perfusion with buffer I (NaCl 120.4 mmol/L, KCl 14.7 mmol/L, NaHCO_3_ 0.6 mmol/L, KH_2_PO_4_ 0.6 mmol/L, Na_2_HPO_4_ 0.4 mmol/L, MgSO_4_ 1.2 mmol/L, HEPES 10 mmol/L, taurine 30 mmol/L, 2,3-butanedione monoxime 10 mmol/L, glucose 5.5 mmol/L), the buffer was switched to buffer II, buffer I supplemented with CaCl_2_ 12.5 μmol/L, collagenase B (Roche) 0.32 mg/mL, collagenase D (Roche) 0.24 mg/mL and protease type XIV (Sigma) 0.004 mg/mL. For protein samples, NaF 1 mmol/L and Na_3_O_4_V 1 mmol/L were added to the buffers to prevent dephosphorylation. After 20 min of perfusion, cells were isolated through tearing the heart tissues with fine forceps and pipetting. Cell suspensions were subsequently filtered through 200 μm nylon mesh to remove undigested tissue debris. Flow-through suspensions were then filtered through a 40 μm cell strainer and the fraction which remained on the 40 μm cell strainer was considered a cardiomyocyte-rich fraction. By this isolation strategy, cardiomyocytes were substantially enriched (pre-isolation: 39.7%, post-isolation: 78.0%)^63^.

### Cardiomyocyte nuclei count

Cardiomyocytes isolated from hearts were suspended in buffer I and seeded onto poly-L-lysine-coated glasses. Five min of centrifugation at 200 G reinforced adherence of the cells to the glasses. After fixation with 4% PFA for 15 min, anti-α-actinin primary antibody at 1/400 was incubated with the cells, followed by Alexa Fluor 488-conjugated secondary antibody at 1/400 and DAPI. The number of nuclei in single cardiomyocytes was counted by a researcher who was blinded to the experimental condition.

### Cardiomyocyte fate mapping

*α-MHC-MerCreMer* mice were a generous gift from Dr. Jeffery Molkentin, Cincinnati Children’s Hospital Medical Center and Howard Hughes Medical Institute. *α-MHC-MerCreMer* mice and *Rosa26-STOP-eYFP* mice obtained from Jackson Laboratories were back-crossed onto BALB/c background for eight generations and mated to label pre-existing cardiomyocytes with eYFP by tamoxifen injection. Tamoxifen (Sigma) (0.5 mg/body/day) was intraperitoneally injected for 14 consecutive days, followed by 5 days of no-treatment period before EAM induction.

### Immunoblotting

Immunoblotting was performed as described previously^18^. Antibodies were used at 1/500 and are listed in Supplementary Table S2.

### Quantitative RT-PCR

Quantitative RT-PCR was performed according to manufacturer’s protocol. In brief, total RNA was prepared from homogenized tissues or isolated cardiomyocytes using QIAzol Lysis Reagent (Qiagen). Complementary DNA was synthesized from the total RNA and used for real-time RT-PCR analysis (StepOne Real-Time PCR systems, Applied Biosystems) to quantify mRNA expression. The amplification unit comprised Fast SYBR Green Master Mix (Applied Biosystems) and the primers listed in Supplementary Table S3.

### Cardiomyocyte-specific STAT3 conditional knockout mice

*STAT3* flox mice were a generous gift from Dr. Kiyoshi Takeda, Graduate School of Medicine, Osaka University. The *STAT3* flox mice were back-crossed onto BALB/c background for eight generations and mated with the *α-MHC-MerCreMer* mice. Cardiomyocyte-specific STAT3 conditional knockout was achieved by intraperitoneal injection of tamoxifen (0.5 mg/body/day) for 14 consecutive days, followed by 5 days of no-treatment period before EAM induction.

### Echocardiography

Mice were anesthetized with isoflurane and subjected to echocardiography on M-mode using an iE33 model equipped with a 15-MHz transducer (Philips Electronics, Andover, MA). The investigator was blinded to the identity of the mice.

### Microarray analysis

Total RNA was prepared from cardiomyocytes that were isolated from STAT3fl/fl and STAT3cKO hearts at EAM3w. Total RNA, pooled from four STAT3fl/fl mice and four STAT3cKO mice, was used for microarray analysis as control and test samples, respectively. The comprehensive expression comparison was performed by Filgen, Inc. using GeneChip® Mouse Gene 2.0 ST Array (Affymetrix).

### Statistics

Comparison between two groups was performed with Welch’s *t* test or Mann Whitney *U* test. For multiple groups with normal distribution and equal variance, one-way ANOVA or two-way ANOVA was applied, followed by *post-hoc* tests. Multiple groups with non-normal distribution and/or different variance were compared with Kruskal-Wallis test or Steel-Dwass test. The statistical significance level was set at *P* < 0.05. Data were presented as mean ± s.d.

## Electronic supplementary material

Supplementary Information

Dataset 1
