# Supplementary material for: Adult murine cardiomyocytes exhibit regenerative activity with cell cycle reentry through STAT3 in the healing process of myocarditis
Source: Sci Rep. 2017 May 3;7:1407. doi: 10.1038/s41598-017-01426-8 (PMC5431117; doi:10.1038/s41598-017-01426-8)
Supplement: Supplementary file 1 — Supplementary Information [file 41598_2017_1426_MOESM1_ESM.pdf]

Adult murine cardiomyocytes exhibit regenerative activity with cell cycle reentry through STAT3 in the healing process of myocarditis

Akimitsu Miyawaki, Masanori Obana, Yusuke Mitsuhara, Aya Orimoto, Yusuke Nakayasu, Tomomi Yamashita, So-ichiro Fukada, Makiko Maeda, Hiroyuki Nakayama, Yasushi Fujio.

Supplementary materials:

Supplementary methods

Supplementary Figure S1. Cardiac tissue was restored at EAM5w
Supplementary Figure S2. Cardiomyocytes were efficiently labeled with eYFP
Supplementary Figure S3. STAT3 was activated at EAM3w
Supplementary Figure S4. STAT3 activation was attenuated at EAM5w
Supplementary Figure S5. Cardiomyocyte STAT3 was deleted in STAT3cKO mice
Supplementary Figure S6. Cardiac tissue restoration was impaired in STAT3cKO mice
Supplementary Figure S7. Capillary density was unaltered in STAT3cKO hearts
Supplementary Figure S8. STAT3 protected cardiomyocytes in the inflamed heart
Supplementary Figure S9. IL-11 administration enhanced cardiomyocyte proliferation at EAM3w
Supplementary Figure S10. Rigid collagen fiber was observed in the injured area at EAM5w
Supplementary Figure S11. Meis1 expression was unchanged in EAM model

Supplementary Table S1. Echocardiographic analysis of STAT3fl/fl and STAT3cKO mice
Supplementary Table S2. Antibodies
Supplementary Table S3. Primer sequences

Supplementary methods

Capillary density quantification

Heart sections were fixed with acetone for 15 min. The endogenous peroxidase was silenced with 0.3% H_2O_2 in methanol, followed by blocking with 3% bovine serum albumin (BSA). Anti-CD31 primary antibody and biotin-linked secondary antibody were applied at 1/200 in sequence. Vectastain ABC kit (Vector Laboratories) and DAKO® Liquid DAB + (DAKO) were used for staining. The number of $CD31^+$ cells was counted by a researcher who was blinded to the experimental condition, and capillary density at post-inflamed regions was calculated by dividing the number by the area of post-inflamed regions.

IL-11 administration

IL-11 (0.5 μg in 200 μL PBS) was intravenously administered in mice at EAM3w, followed by four times of intraperitoneal BrdU administration. The heart sections were collected 24 hours after the last BrdU injection and subjected to immunofluorescence staining.

Picrosirius red staining

Picrosirius red staining was performed using Picro-Sirius Red Stain Kit (Scytek) according to manufacturer's protocol. Briefly, frozen heart sections were fixed with 4% PFA for 15 min. After rinsing in distilled water, the sections were incubated with Picro-sirius Red Solution for 60 min, followed by two quick rinses in 0.5% Acetic Acid Solution. Dehydration in absolute ethanol was performed prior to clearing and mounting.

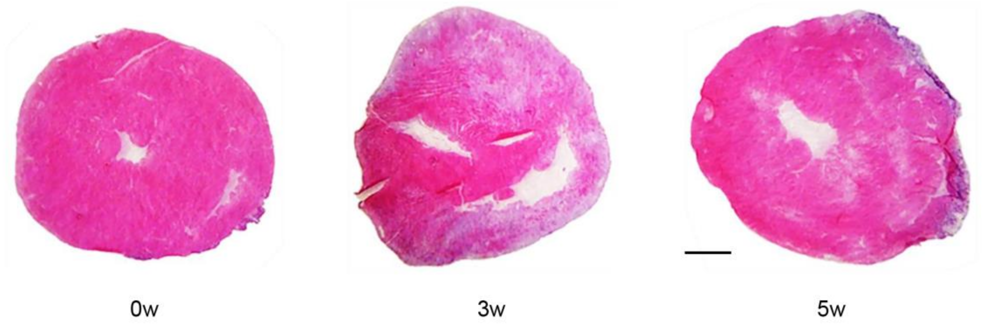

Supplementary Figure S1. Cardiac tissue was restored at EAM5w.

The entire HE-stained cross sections of EAM0w, EAM3w and EAM5w hearts are shown. Scale bar: 1 mm.

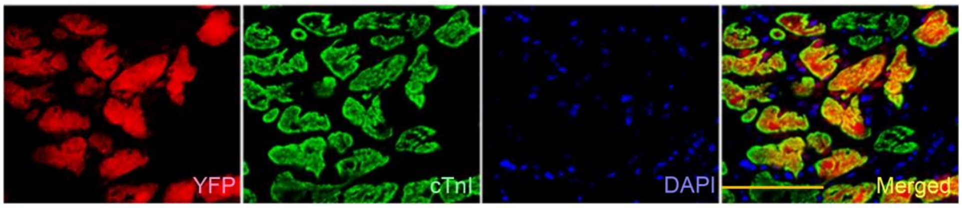

Supplementary Figure S2. Cardiomyocytes were efficiently labeled with eYFP. Cardiomyocytes were labeled with eYFP in advance to EAM induction, as described in Methods. Heart sections were immunostained for cTnI and YFP at EAM3w. Scale bar: 50 μm.

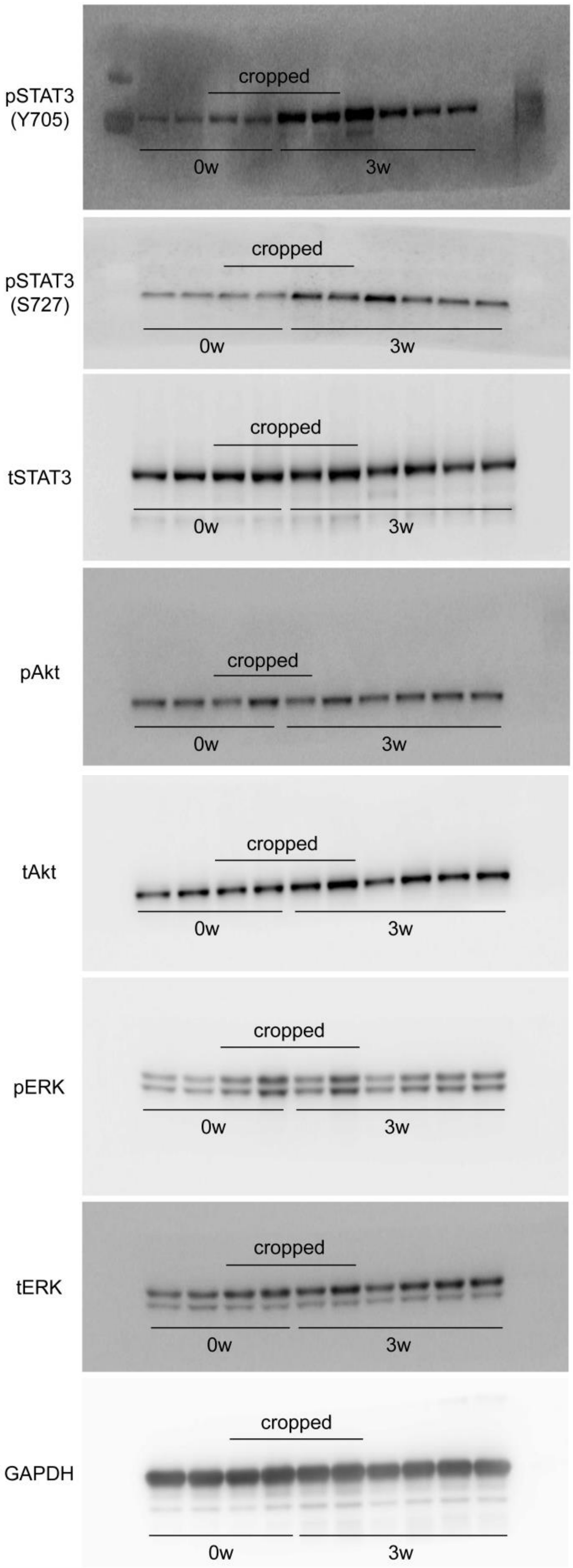

Supplementary Figure S3. STAT3 was activated at EAM3w.

The full-length blots of Figure 4a are shown.

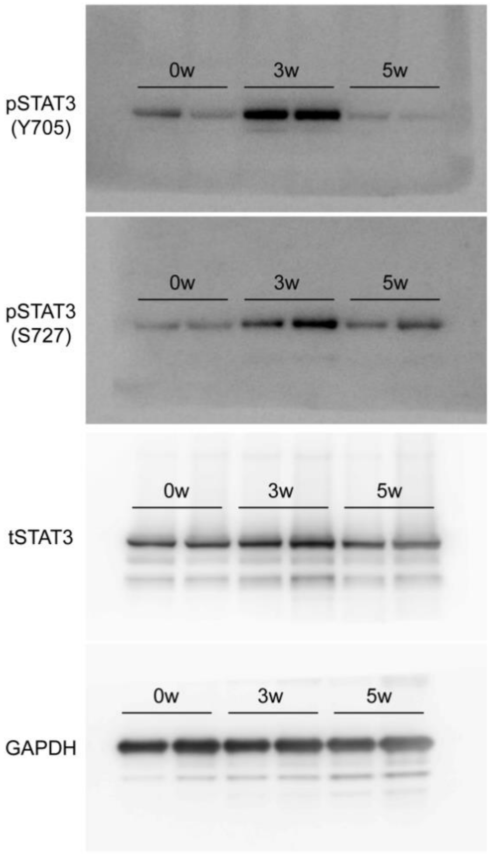

Supplementary Figure S4. STAT3 activation was attenuated at EAM5w.

Heart homogenates at EAM0w, EAM3w and EAM5w were subjected to immunoblotting with anti-pSTAT3 (Y705), anti-pSTAT3 (S727), anti-tSTAT3 and anti-GAPDH antibodies.

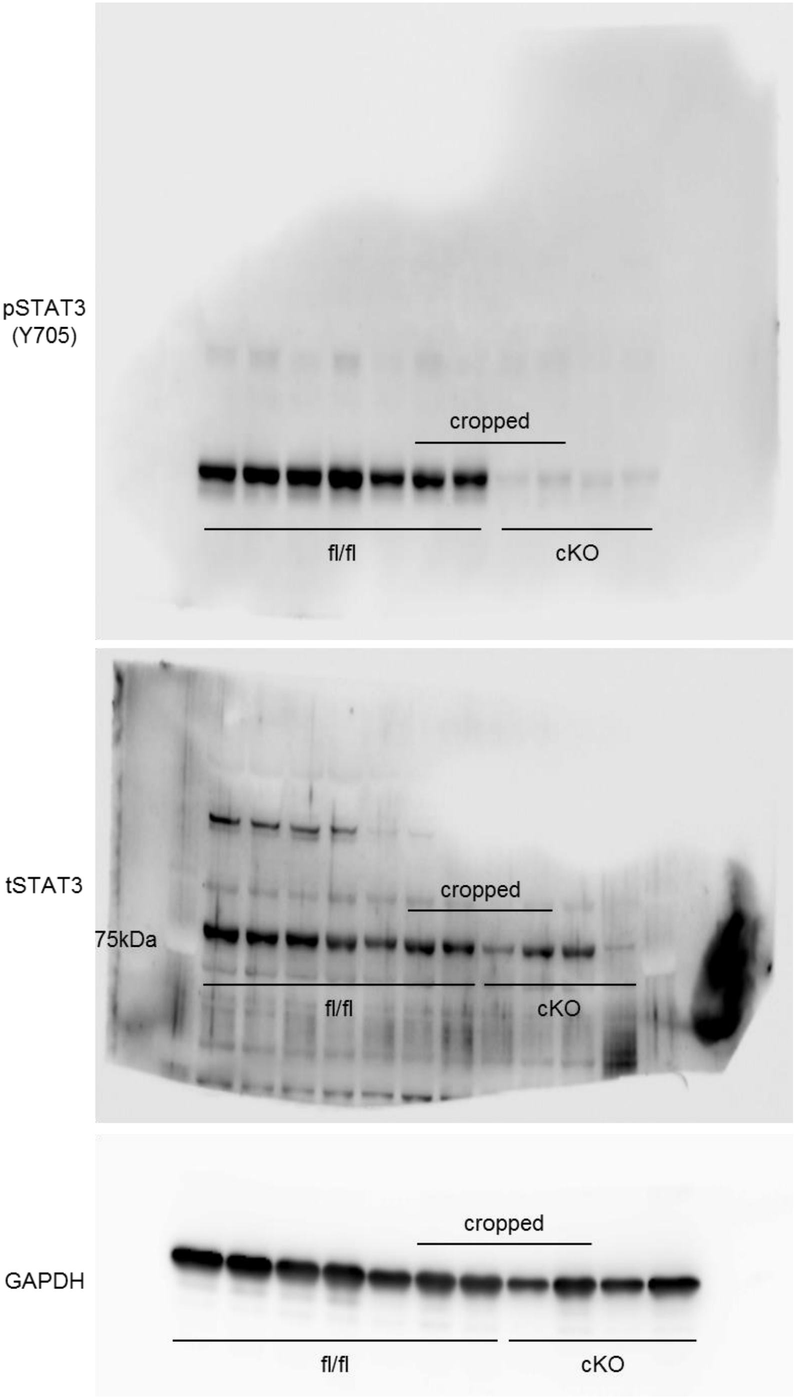

Supplementary Figure S5. Cardiomyocyte STAT3 was deleted in STAT3cKO mice.
The full-length blots of Figure 5b are shown.

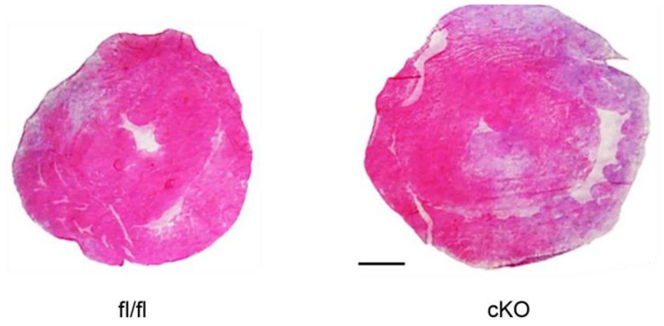

Supplementary Figure S6. Cardiac tissue restoration was impaired in STAT3cKO mice. The entire HE-stained cross sections of STAT3fl/fl and STAT3cKO hearts at EAM5w are shown. Scale bar: 1 mm.

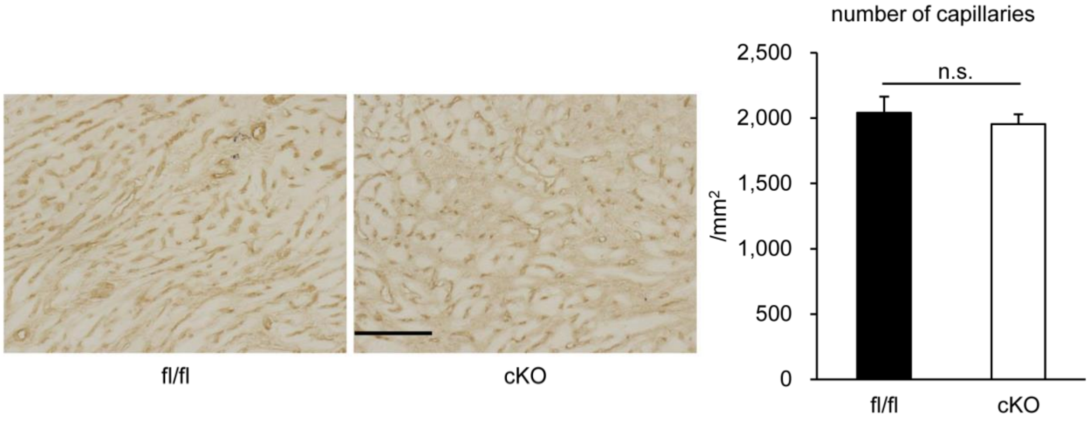

Supplementary Figure S7. Capillary density was unaltered in STAT3cKO hearts. Immunohistochemical analysis with anti-CD31 antibody was performed for STAT3fl/fl and STAT3cKO heart sections at EAM5w. Left: representative images are shown. Scale bar: 100 μm. Right: $CD31^+$ cells were counted in number and capillary density was calculated. n=7 mice for each group. Welch's *t*-test. Data are shown as mean ± s.d.

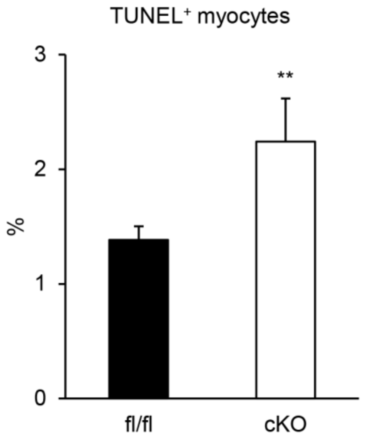

Supplementary Figure S8. STAT3 protected cardiomyocytes in the inflamed heart. TUNEL staining was performed for heart sections of STAT3fl/fl and STAT3cKO mice at EAM3w. $TUNEL^{+}MHC^{+}$ cells in the inflamed region were counted and shown as percentage in MHC^{+} cells. n=4 mice for fl/fl; 6 mice for cKO. Welch's *t*-test. $^{**}P<0.01$. Data are shown as mean ± s.d.

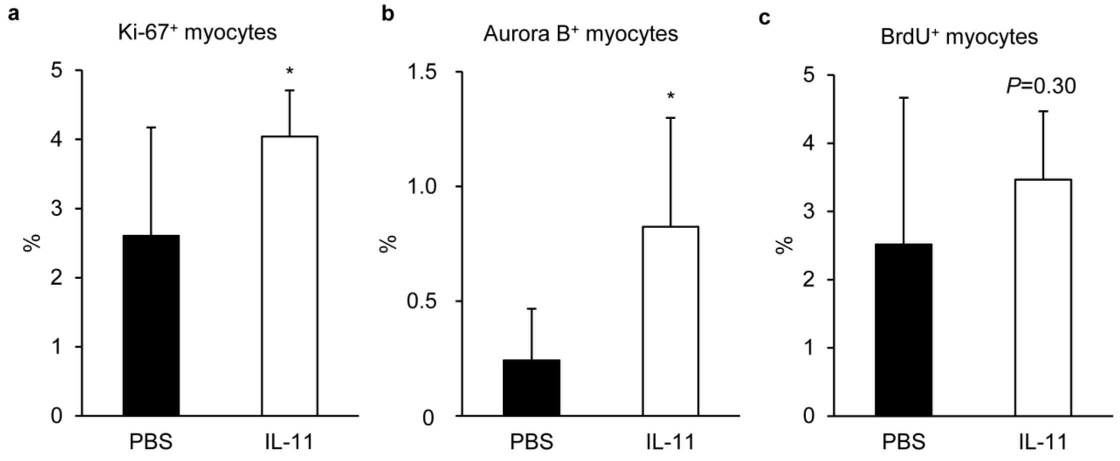

Supplementary Figure S9. IL-11 administration enhanced cardiomyocyte proliferation at EAM3w.

(**a**) Heart sections from PBS- or IL-11-treated mice at EAM3w were immunostained for Ki-67 and MHC. Ki-67^{+}MHC^{+} cells in the inflamed region were counted and the percentage of Ki-67^{+}MHC^{+} cells in MHC^{+} cells is shown. n=8 mice for PBS; 6 mice for IL-11. (**b**) Heart sections from PBS- or IL-11-treated mice at EAM3w were immunostained for Aurora B and cTnI. Aurora B^{+}cTnI^{+} cells in the inflamed region were counted and the percentage of Aurora B^{+}cTnI^{+} cells in cTnI^{+} cells is shown. n=8 mice for PBS; 6 mice for IL-11. (**c**) BrdU incorporation assay was performed for PBS- or IL-11-treated mice at EAM3w. Heart sections were immunostained for BrdU and MHC 24 hours after the last BrdU injection. BrdU^{+}MHC^{+} cells in the inflamed region were counted and the percentage of BrdU^{+}MHC^{+} cells in MHC^{+} cells is shown. n=8 mice for PBS; 6 mice for IL-11. Welch's *t*-test. $^{*}P<0.05$. Data are shown as mean ± s.d.

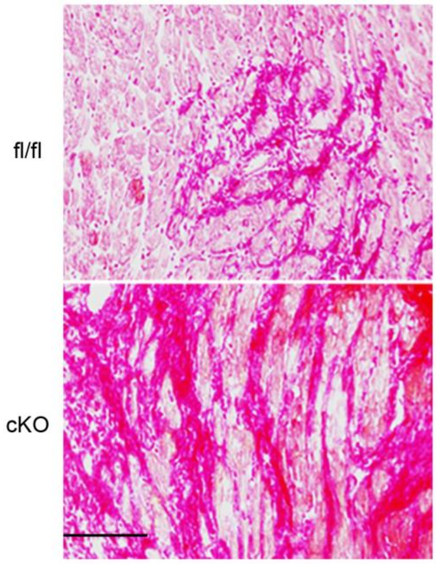

Supplementary Figure S10. Rigid collagen fiber was observed in the injured area of STAT3cKO hearts at EAM5w.

Picrosirius red staining was performed for heart sections of STAT3fl/fl and STAT3cKO mice at EAM5w. Scale bar: 100 μm.

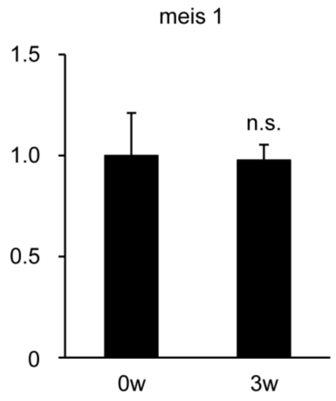

Supplementary Figure S11. Meis1 expression was unchanged in EAM model.

Cardiomyocytes were isolated at EAM0w and EAM3w, and meis1 mRNA expression was quantified by quantitative RT-PCR. The expression of meis1 was normalized to that of gapdh and shown as fold increase relative to 0w. n=7 mice for 0w; 6 mice for 3w. Welch's *t*-test. Data are shown as mean ± s.d.

Supplementary Table S1. Echocardiographic analysis of STAT3fl/fl and STAT3cKO mice.

EAM	0w		5w	
Genotype	fl/fl	cKO	fl/fl	cKO
FS (%)	48.6 ± 4.8	45.9 ± 8.6	44.2 ± 6.7	35.0 ± 8.5*
LVIDd (mm)	3.5 ± 0.3	3.5 ± 0.4	3.4 ± 0.3	3.6 ± 0.5
LVIDs (mm)	1.8 ± 0.3	1.9 ± 0.4	1.9 ± 0.4	2.4 ± 0.5
IVSd (mm)	0.9 ± 0.1	0.9 ± 0.1	0.9 ± 0.1	0.9 ± 0.1
LVPWd (mm)	0.7 ± 0.1	0.7 ± 0.1	0.8 ± 0.1	0.8 ± 0.1
HR (bpm)	469.4 ± 84.1	446.7 ± 45.9	468.0 ± 64.2	441.3 ± 66.2

FS: fractional shortening, LVIDd: left ventricular internal dimension in diastole, LVIDs: left ventricular internal dimension in systole, HR: heart rate, IVSd: interventricular septum thickness in diastole, LVPWd: left ventricular posterior wall thickness in diastole, HR: heart rate. Data are shown as mean ± s.d. n=9 mice for each group. Two-way repeated measures ANOVA. *P<0.05 vs cKO 0w.

Supplementary Table S2. Antibodies.

Target	Manufacturer	Cat. #
MHC	Santa Cruz	sc-20641
cTnI	Santa Cruz	sc-8118
Ki-67	eBioscience	14-5698-80
Aurora B	Abcam	ab2254
BrdU	Abcam	ab6326
α-actinin	Sigma	A7811
YFP	MBL	598
	SICGEN	AB0020-200
pSTAT3 (Y705)	Abcam	ab76315
	Cell Signaling	9131
pSTAT3 (S727)	Cell Signaling	9134
tSTAT3	Cell Signaling	4904
pAkt	Cell Signaling	9271
tAkt	Cell Signaling	9272
pERK	Cell Signaling	9101
tERK	Cell Signaling	9102
GAPDH	Millipore	MAB374
CD11b	BD Pharmingen	550282
vimentin	Santa Cruz	sc-7558-R
CD31	BD Pharmingen	553370

Supplementary Table S3. Primer sequences.

Gene	Direction	Sequence
gapdh	forward	5'-CATCACCATCTTCCAGGAGCG-3'
	reverse	5'-GAGGGGCCATCCACAGTCTTC-3'
IL-6	forward	5'-AAGAGACTTCCTTCCAGTTGCCTTC-3'
	reverse	5'-ATTATATCCAGTTTGGTAGCATCCATC-3'
IL-11	forward	5'-CCATGAGCGCTGGGACATTG-3'
	reverse	5'-TAGGGAAGGACCACCTGCAC-3'
LIF	forward	5'-TCCTATTACACAGCTCAAGG-3'
	reverse	5'-ACTTGCTTGTATGTCCCCAG-3'
OSM	forward	5'-GAATCAGGCGAACCTCACG-3'
	reverse	5'-GCATCCAGTTGGTACAAGACTC-3'
CLCF1	forward	5'-TACCTGGAGCATCAACTCCG-3'
	reverse	5'-CGCCACACTTCCAAGTTGAC-3'
CT-1	forward	5'-TCTATGGCGAGTGGGTGAGC-3'
	reverse	5'-AGCAAGCAAGCAAAGAAAGA-3'
IL-1β	forward	5'-GACAAAATACCTGTGGCCTTGGGCC-3'
	reverse	5'-GAGGTGCTGATGTACCAGTTGGGGA-3'
IL-17A	forward	5'-TTTAACTCCCTTGGCGCAAAA-3'
	reverse	5'-CTTTCCCTCCGCATTGACAC-3'
TNF-α	forward	5'-CCATTCCTGAGTTCTGCAAAGG-3'
	reverse	5'-AGGTAGGAAGGCCTGAGATCTTATC-3'
TGF-β	forward	5'-AGAGGTCACCCGCGTGCTAA-3'
	reverse	5'-TCCCGAATGTCTGACGTATTGA-3'
collagen type I	forward	5'-GCGAGTGCTGTGCTTTCTG-3'
	reverse	5'-TCCCTCGACTCCTACATCTTC-3'
collagen type III	forward	5'-ATGCCCACAGCCTTCTACAC-3'
	reverse	5'-CCCAGGGTCACCATTTCTCC-3'
cyclin D1	forward	5'-GCGTACCCTGACACCAATCTC-3'
	reverse	5'-CTCCTCTTCGCACTTCTGCTC-3'
cyclin D2	forward	5'-GAGTGGGAACTGGTAGTGTTG-3'
	reverse	5'-CGCACAGAGCGATGAAGGT-3'
cyclin D3	forward	5'-CGAGCCTCCTACTTCCAGTG-3'
	reverse	5'-GGACAGGTAGCGATCCAGGT-3'
cyclin E	forward	5'-GTGGCTCCGACCTTTCAGTC-3'
	reverse	5'-CACAGTCTTGTCAATCTTGGCA-3'
cyclin A	forward	5'-TGGATGGCAGTTTTGAATCACC-3'
	reverse	5'-CCCTAAGGTACGTGTGAATGTC-3'

cyclin B1	forward	5'-ATGCTGGACTACGACATGGTG-3'
	reverse	5'-ACTGTAGGATAGGTAGTGCTGC-3'
cdk2	forward	5'-CCTGCTTATCAATGCAGAGGG-3'
	reverse	5'-GTGCTGGGTACACACTAGGTG-3'
cdk4	forward	5'-ATGGCTGCCACTCGATATGAA-3'
	reverse	5'-TCCTCCATTAGGAACTCTCACAC-3'
cdk6	forward	5'-GGCGTACCCACAGAAACCATA-3'
	reverse	5'-AGGTAAGGGCCATCTGAAAACT-3'
MT1	forward	5'-CGTAGCTCCAGCTTCACCAGATCTC-3'
	reverse	5'-TGGTGGCAGCGCTGTTCGT-3'
MT2	forward	5'-GCTTTTGCGCTCGACCCAATACTCTC-3'
	reverse	5'-GGAGCAGCAGCTTTTCTTGCAGGAAG-3'
clusterin	forward	5'-AGCAGGAGGTCTCTGACAATG-3'
	reverse	5'-GGCTTCCTCTAAACTGTTGAGC-3'
meis1	forward	5'-TGATGACACGGCATCCACTCG-3'
	reverse	5'-CCAAGCCATCACCTTGCTCAC-3'
